# Accurate image-based CSF volume calculation of the lateral ventricles

**DOI:** 10.1038/s41598-022-15995-w

**Published:** 2022-07-15

**Authors:** Fernando Yepes-Calderon, J. Gordon McComb

**Affiliations:** 1 Science Based Platforms LLC, R&D, 604 Beach CT, Fort Pierce, 34950 USA; 2 Division of Neurosurgery, Children Hospital Los Angeles, Los Angeles , 90027 USA; 3 GYM Group SA, R&D, Carrera 78A 6-58, Cali, Valle del Cauca 76001 Colombia; 4 Keck School of Medicine, University of Southern California, Los Angeles, 90033 USA

**Keywords:** Neuroscience, Paediatric research, Biomedical engineering

## Abstract

The size/volume of the brain’s ventricles is essential in diagnosing and treating many neurological disorders, with various forms of hydrocephalus being some of the most common. Initial ventricular size and changes, if any, in response to disease progression or therapeutic intervention are monitored by serial imaging methods. Significant variance in ventricular size is readily noted, but small incremental changes can be challenging to appreciate. We have previously reported using artificial intelligence to determine ventricular volume. The values obtained were compared with those calculated using the inaccurate manual segmentation as the “gold standard”. This document introduces a strategy to measure ventricular volumes where manual segmentation is not employed to validate the estimations. Instead, we created 3D printed models that mimic the lateral ventricles and measured those 3D models’ volume with a tuned water displacement device. The 3D models are placed in a gel and taken to the magnetic resonance scanner. Images extracted from the phantoms are fed to an artificial intelligence-based algorithm. The volumes yielded by the automation must equal those yielded by water displacement to assert validation. Then, we provide certified volumes for subjects in the age range (1–114) months old and two hydrocephalus patients.

## Introduction

The development of MRI/CT has revolutionized the ability to visualize the *Central Nervous System* (CNS)^1,2^. With progressively more capabilities being added, one can note structures in greater detail and track changes taking place in the CNS^3,4^. One area of considerable interest is *Cerebrospinal Fluid* (CSF) volume within the cranium^5–8^. As a distinct boundary exists between CSF spaces and parenchyma, it is possible to determine the CSF volume, especially within the lateral ventricles, to note differences in ventricle size in response to disease progression and therapeutic intervention both acutely and over a protracted-time period^9,10^. We developed a segmentation strategy based on multiple algorithms utilizing MRI images to determine ventricular volume, which was compared with volume determined by manual segmentation^11^. Manual segmentation is tedious, time consuming and operator dependent^12,13^. Therefore, we started considering a better way to calculate the lateral ventricular volume avoiding manual segmentation-the current study documents how we have been able to achieve the goal.

This paper proposes an electronic device that determines volume from the 3D printed models using light sensors. We employed the proposed method to measure the ventricular volumes in children with ages in [1, 6, 15, 24, 48, 66, 78, 96, 114] months old and two *hydrocephalus (HC)* cases. The obtained volumes are displayed for clinical use.

## Results

### Tuning the water-displacement (WD) instrument

The Tables 1 and 2 should be observed together. In the tuning tasks, we obtained the real volume (Real Vol) value analytically using the marble diameter. The associated uncertainty is related to the propagation of the caliper’s precision. The records on these tables correspond to calculations done on the individual marbles. Bigger volumes can be reached by submerging several marbles per experiment.

During the tuning process, one can calculate the flux per slot produced by the flow sensor thanks to the object’s analytically estimated volume (Real Vol in the tables). After measuring several marbles and marbles’ aggregation, we created a look-up table that returns a flux per slot when the slot time is given.

**Table 1:** Marbles’ volumes, pulse counting in the flux sensor and estimated fluxes. Tuning bigger volumes requires marbles aggregation ($Ma_{1} + Ma_{2} +\cdots Ma_{n}$) and the supervisor factor (real vol), is found by adding the analytically estimated volumes of the submerged marbles.

Count (pulses)	Model	Real vol (ml)	Mean flux (ml/s)	Mean flux/slot (ml)
61	Ma1	$8.1\pm 1.0$	$4.4\pm 0.5$	0$.133\pm 0.016$
58	Ma2	$8.0\pm 1.0$	$4.5\pm 0.5$	0$.138\pm 0.017$
66	Ma3	$7.3\pm 0.9$	$3.5\pm 0.4$	0$.111\pm 0.014$
65	Ma4	$7.6\pm 0.9$	$3.7\pm 0.5$	0$.117\pm 0.014$
63	Ma5	$7.9\pm 1.0$	$4.1\pm 0.5$	0$.125\pm 0.015$
252	Ma6	$35.6\pm 0.9$	$4.7\pm 0.04$	0$.141\pm 0.001$
474	Ma7	$60.1\pm 0.9$	$4.4\pm 0.03$	0$.135\pm 0.001$

### Volume measurements on 3D objects

**Table 2:** Marbles’ volumes and time slot statistics per experiment.

Count (pulses)	Model	Real vol (ml)	Max time	Mean time	Min time	Std time
61	Ma1	$8.1\pm 1.0$	0.037157	0.030073	0.027922	0.000779
58	Ma2	$8.0\pm 1.0$	0.037157	0.030398	0.025835	0.000993
66	Ma3	$7.3\pm 0.9$	0.037157	0.031326	0.027922	0.000707
65	Ma4	$7.6\pm 0.9$	0.037157	0.031458	0.027922	0.001300
63	Ma5	$7.9\pm 1.0$	0.037157	0.030483	0.027922	0.000795
252	Ma6	$35.6\pm 0.9$	0.033232	0.029899	0.025774	0.000930
474	Ma7	$60.1\pm 0.9$	0.034960	0.030765	0.026517	0.000837

The differences in the time per pulse, namely time stats in Table 2, suggest an irregular operation in the pumping device that tends to stabilize itself when the pump is operative for extended periods. During volume estimation, the look-up table created in the WD tuning process, is recurrently queried with slot timings to retrieve the displaced water in milliliters of any irregular submerged object.

See in Table 3, the water displaced by the created 3D replicas of the lateral ventricles.

**Table 3:** Records of reading volumes in physical 3D models by water displacement using the device previously tuned with the marbles. We thoroughly justify the use of sinkers in Section: “The WD device operation”.

Structure	Est vols (ml)	Sinker used	Vols—sinker (ml)
Sinker 1	$13.9\pm 0.8$	–	–
Sinker 2	$12.8\pm 0.6$	–	–
v1mo	$17.3\pm 0.4$	1	$3.4\pm 0.9$
v6mo	$21.2\pm 0.4$	1	$7.3\pm 0.9$
v24mo	$24.4\pm 0.5$	1	$10.5\pm 0.9$
v15mo	$24.7\pm 0.5$	1	$10.8\pm 0.9$
v48mo	$27.2\pm 0.6$	1	$13.3\pm 1.0$
v66mo	$21.9\pm 0.5$	1	$8.0\pm 0.9$
v78mo	$25.4\pm 0.5$	1	$11.5\pm 1.0$
v96mo	$24.8\pm 0.5$	1	$11.0\pm 0.9$
v114mo	$33.5\pm 0.7$	1	$19.7\pm 1.1$
Hydrocephalus moderate	$114.5\pm 0.4$	1,2	$88.4\pm 0.9$
Hydrocephalus severe	$142.0\pm 0.4$	1,2	$115.9\pm 0.9$

### Algorithm validation and measures on original medical images

Once the physical volumes are estimated (Vols—Sinker column), the results are compared to the volumes estimated by the automatic ventricular volume estimator (AVVE)^11^. Note how the automatic method is presented without uncertainty; this happens because the AVVE, unlike the manual assessments, yields reproducible results.

Since the AVVE acting on the scanned phantoms produced values within the range of uncertainty of the WD device, the algorithm is validated. At this point, the validated AVVE is used on original images to produce reliable measurements. See the results of the validating stage and the measurements on original images in Table 4.

**Table 4:** The column WD device(ml) is the gold-standard volume.

Structure	Validation process		Certified AVVE on original images (ml)
	WD device (ml)	AVVE phantom (ml)	
v1mo	$3.4\pm 0.9$	3.3	3.1
v6mo	$7.3\pm 0.9$	7.3	6.8
v24mo	$10.5\pm 0.9$	10.4	9.8
v15mo	$10.8\pm 0.9$	10.9	10.0
v48mo	$13.3\pm 1.0$	13.5	12.5
v66mo	$8.0\pm 0.9$	8.2	9.4
v78mo	$11.5\pm 1.0$	11.0	11.9
v96mo	$11.0\pm 0.9$	10.8	11.4
v114mo	$19.7\pm 1.1$	20.0	21.8
HC moderate	$88.4\pm 0.9$	90.1	99.1
HC severe	$115.9\pm 0.9$	116.7	123.7

The column AVVE phantom(ml) is the volume in the phantoms measured with the AVVE. These two columns are displayed in a box to depict the validation proccess. . Since the AVVE measured within the uncertainty of the WD device, it is certified to measure on the original images and we reported those values for clinical use.

## Discussion

In a previous study, we used multiple algorithms to determine ventricular volume calibrated against that determined by manual segmentation^11^. As manual segmentation is operator-dependent, inaccurate measurements introduce error to the degree that is significant (study to be reported). Also, the method is very tedious, time-consuming, and rarely done except for research purposes. The initial step is to use MRI images and generate 3D printed models of the lateral ventricles whose volume is determined by a precise water displacement method. We assure that the device measures precisely by testing the WD device with analytically known volumes; therefore, the results yielded by automation such as the AVVE could be tested and adjusted with a more reliable tool than manual segmentation.

The tuning procedure can be used repeatedly and only needs to be checked at given intervals for certification.

The strategy requires bigger measuring recipients for bigger volumes, which is a limitation since changing the capacity of measuring recipients also changes the system’s sensitivity. Ideally, one should cover the measuring range without changing the setup.

The presented ventricular volumes for children and the methodology used have a two-folding purpose. To provide the health care community with reference volume-age matched values that are estimated with an method that eliminates uncertainty. To present an reproducible strategy to validate the outcome of quantifying algorithms such as the AVVE.

The whole process is done at the voxel level, decrypted and uncompressed, providing analytic capabilities within the Picture Archive and Communication System (PACS) eliminating the constraints imposed by Health Insurance Portability and Accountability Act (HIPAA) regulation^14^. The last step is to incorporate the ventricular volume into the radiology network to add it to the final report automatically.

## Methods

The retrospective-data gathering and the methods presented in this document strictly comply with relevant guidelines and regulations. This study was approved by the Institutional Review Board of the Children Hospital Los Angeles, which waived the requirement for informed consent because of the retrospective nature of the gathered data. Please, refer to IRB number CHLA-15-00161.

### Medical imaging on neonates and children

Sixty-five (65) MRI clinical studies of children acquired in a Phillips Achieva scanner using gradient recalled with variant configured in steady-state on a B-FFE sequence were gathered from the Children Hospital Los Angeles database. With this imaging configuration, T1 contrast provides an increased signal intensity from fluid while retaining tissue visibility. We did not register imaging acquisition parameters such as TE and TR, as neither of the image geometries, such as matrix size and resolution due to the diverse range of values found in the clinical data. We anonymized the medical data that comprised subjects aged 1 to 114 months broken down into nine age segments, each comprising seven subjects. Calculations on two more infants with hydrocephalus (HC) complement the research.

### Children’ templates

Seven subjects per age segment underwent the templating procedure. Template creation consists of iteratively averaging *n* number of subjects to create an image with a better signal-to-noise ratio than any single contributor subject. For this purpose, one selects a target image and refers the contributors—namely moving images—to the former. The images require preprocessing to assess imaging geometrical consistency. Geometrical consistency is accomplished by executing linear and non-linear registrations before averaging. We partially follow the procedure to create age-specific templates presented in^15^. Our automation does not include an external template such as the MNI-152. Instead, the algorithm labels the best signal-to-noise ratio (SNR) subject as the target for each age segment. We computed the SNR between the brain region and the image’s background using FSL-bet^16,17^ for separating the regions. Finally, the algorithm resamples the target study to isometric voxels of $1\;$mm. Subsequently, we execute segmentation tasks on templates with voxel resolution $1\;{\rm mm}^3$ and original resolution for the two hydrocephalus cases.

### Measuring volume with Archimedes’ principle

**Figure 1:**
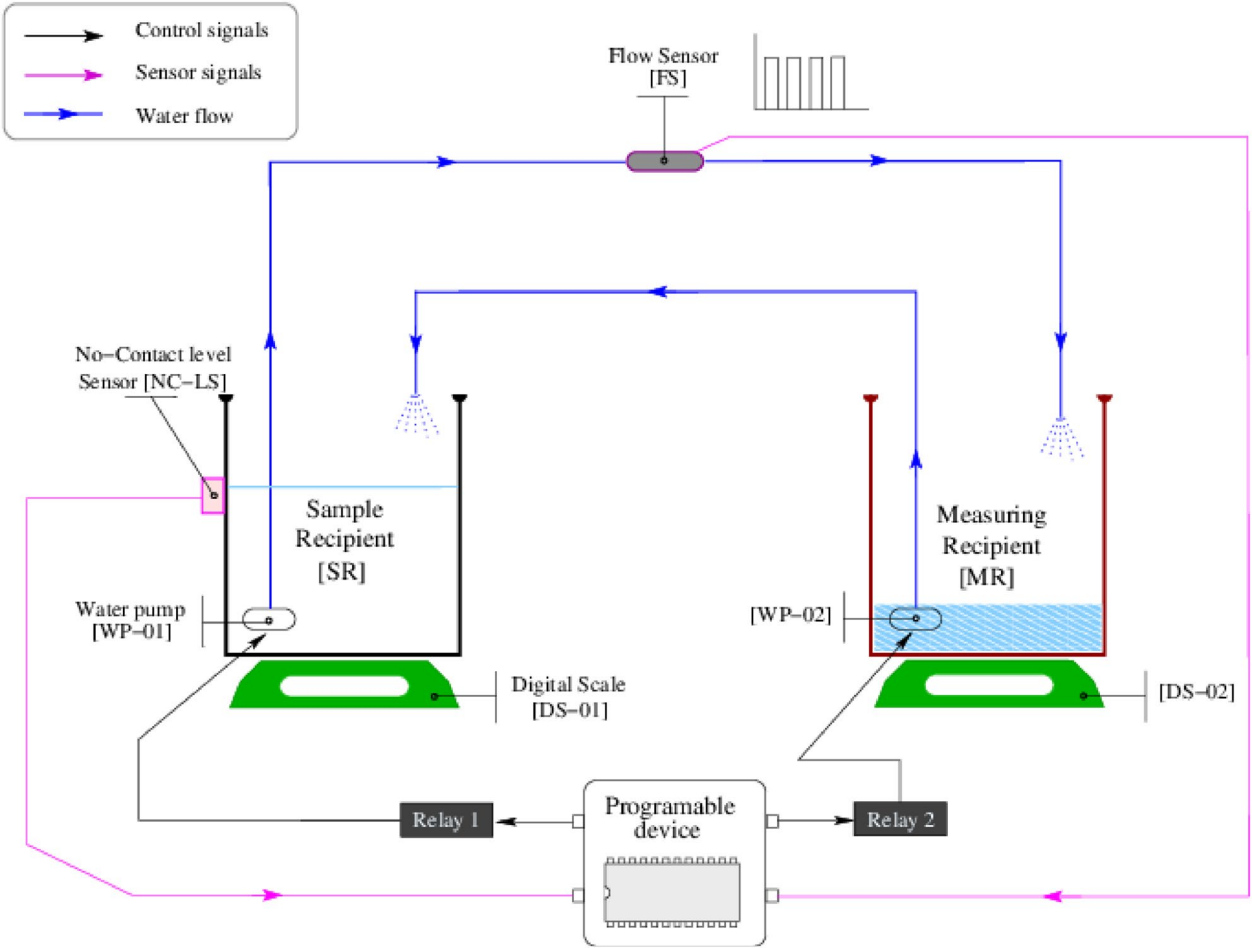
Design of the water-displacement-measuring device.

We chose the water displacement (WD) principle stated by Archimedes^18^ to measure the irregular volumes of the 3D reproduced ventricles. The conceptual design of the device is shown in the Fig. 1.

The electronic device uses a non-contact sensor to control the operation of electric pumps and a flow sensor that traduces the water flux into pulses.

The montage consists of a measuring recipient [MR] and a sample recipient [SR], both hosting electrical water pumps [WP-01] and [WP-02]. The recipients rest on digital scales [DS-01] and [DS-02] with a precision of 1 ml. The [SR] has a non-contact-level sensor [NC-LS], which works as a digital switch. The [NC-LS] is turned on when water reaches or exceeds the sensor level; it is off otherwise. The pumps are connected to two tubes in a way that depleting one recipient fills the other. The tube that drains [SR] connects a flow sensor [FS] that, in turn, produces pulses when the water flows. The [FS] is specified to read fluxes in the range of 0.1–3 L/min. A Beagle Bone Black card (Programmable device) controls the operation of this device, reading Transistor-to-Transistor logic (TTL) signals from its sensor ports (magenta lines) and using the writing on output ports (black lines) to activate/deactivate the pumps over the residential energy distribution (120 V to 60 Hz) through transistorized power interfaces.

### The water displacement device operation

To start, the water level in [SR] is below the [NC-LS] sensor; thus, [NC-LS] sends a 0 through its sensor line. Then [WP-02] is activated to push water on [SR] until the water reaches the [NC-LS] level. At this moment, the programmable device will see a logic 1 in the [NC-LS] sensor line. Next, [WP-01] is activated to deplete water from [SR] to find the zero-level. At that moment, the programmable device sees a zero in the [NC-LS] line. The sample is then submerged in [SR], raising the water level above the [NC-LS] sensor forcing a logic 1 in the sensor line. With a one in the sensor line, the programmable device turns on the [WP-01] and activates the pulse counting in the [FS] sensor line. The water pumping from [SR] will remain actively draining until the water level reaches the zero-level. The volume of the displaced water is equal to the volume of the submerged object, and the pulsating pattern yielded by the [FS] sensor is a numeric representation of the displaced volume. Since the 3D volumes were created with 25% of structural filling, sinkers are needed to eliminate buoyancy.

### Precision estimation and tuning process

One can determine a marble’s volume analytically by measuring the diameter (D). To this purpose, we use a caliper of precision 0.1 mm and formulation $V = \frac{\pi *D^3}{6}$. The volume *V* obtained by formulation serves as a supervisor factor to tune the operation of the WD device. We accurately estimate the WD device’s precision by relating the pulse counting produced by the displaced water of analytically known volumes. We estimate reproducibility and calculate the uncertainty of the created device by performing five-folding WD exercises with marbles covering volumes expected in the ventricles.

**Figure 2:**
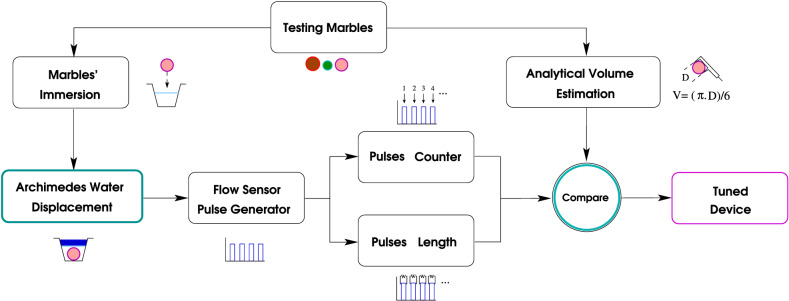
Since the automation presents reproducibility, the tuning process using spheres of known volume as supervisory factor in the range of operation, guaranties the precision of the WD device. One iteration of the tuning process is shown in the Supplementary Video S1.

The WD device tuning process starts with all marbles being labeled following an in-house designed mnemonics. Then, we submerge the tuning marble in [MR], activate the water-displacement device, and save the produced pulsation. Finally, the look-up tables presented in the Section: “Estimating volume from pulses” turn the pulsation into displaced milliliters. These steps are repeated five times with each marble or marble combination. The reported uncertainty *u* is given by $u = (Vmax_{k} - Vmin_{k})/2$, which is the half of the difference between the largest *Vmax* and smallest *Vmin* calculated volumes among the five readings per marble or marble combination *k*. See in Fig. 2 the graphical representation of the tuning pipeline.

### Estimating volume from pulses

**Figure 3:**
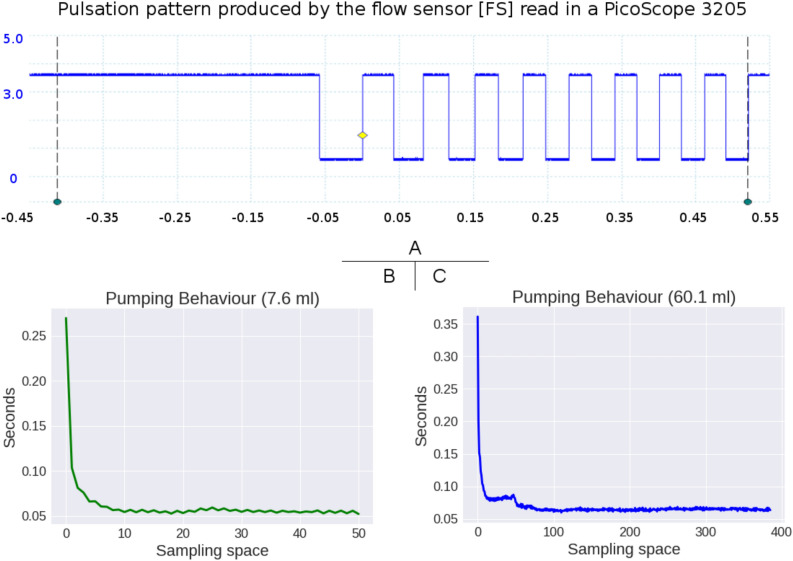
The pulsation pattern of the flow sensor. In (**A**), the signature of the [FS] sensor. In (**B,C**), plots of the timing-slots of two different volumes showing the irregular pumping performance of the [WP-01] device.

In Fig. 3B,C, The pump [WP-01] does not uniformly move the water. The strategy consists of dynamically estimating the variations of flux captured by our system as timing deviations in the pulsating pattern given by [FS]. For this purpose, we have created a tuning routine that consists of measuring all the available volumes and combinations to cover the operating range. The challenge dwells in estimating the flux between consecutive pulses—or slot—in every experiment, so the addition of small contributions per slot reach the known volume (see the pulses in Fig. 3A). In every tuning experiment, we end by having a timing scheme and a flux per slot. We combine all supervised tests’ contributions in two histogram-look-up tables. One table carries the timing classes in their bins, while the other carries fluxes. Further unknown volumes produce timing schemes that we translate to volume through the previously created look-up tables.

In each marble’s timing array $T_{p}$, it is possible to estimate the flux $(F_p[i])$ in the timing slot $(tslot_i)$ by

1 $$\begin{aligned} F_p[i] = \frac{tslot_{i}}{time_{total}} * RV \,\, \forall \, i \in len(T_{p}) \end{aligned}$$

In Eq. (1), *RV* stands for Real Volume and corresponds to the analytically estimated volume of the tuning marble. RV is determined once per marble.

At the end of this process, every timing array $T_{p}$ has an array of fluxes $F_{p}$ associated. Then, let $T_{up}$ be the collection of all available $T_{p}$ and $F_{up}$ the collection of all available estimated fluxes $F_p$.

Now we can create the timing look-up table as follows.

An array of distances $A_d$ is built using $K_{i}$ slots of distances $d = \frac{1}{2^{2j}}$ for $j=[1,1.5,2,2.5,3,3.5]$ and $i = [1,2,2,4,8,16]$. Note how all these fractions of the unity when distributed as ordered by $K_i$ add to 1.

Next, let $m_u$, $t_{min}$ and $t_{max}$ be the mean, min value and max value in $T_{pu}$. From here, the distances *d*1 and *d*2 are calculated as $d_1=m_u - t_{min}$ and $d_2 = t_{max} - m_{u}$.

The timing look-up table $T_{lut}$ is created by concatenating the arrays $A_1 = d_1(A_d)$ and $A_2=d_2(A_d)$.

For the flux look up table, assume $R_{(a,b)}$ to be all indexes *i* where $T[i]_{pu} \in range(a,b)$, where a,b are times in $T_{lut}$, then:

2 $$\begin{aligned} F^{a,b}[i] = \sum F[i]_{up} \,\, \forall \, i \in R_{(a,b)} \end{aligned}$$

When $F^{a,b}$ cover all possible ranges in $T_{lut}$, it becomes $F_{lut}$ or volume displaced by the object.

From this point on, it is possible to estimate any new volume by obtaining its pulsating pattern with the WD device and using the look-up tables $T_{lut}$, $F_{lut}$.

### Creating volume gold standards

**Figure 4:**
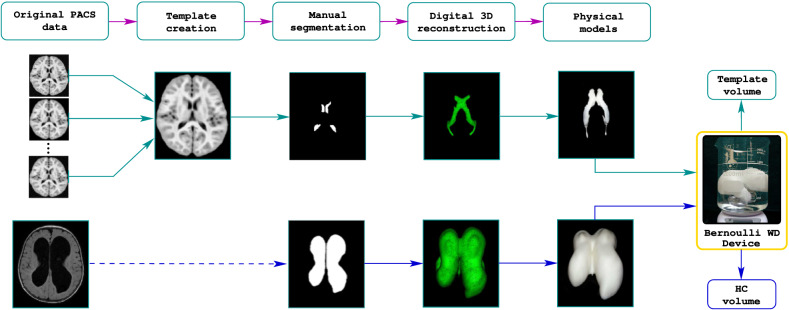
Ventricular volume gold standards creation. From medical images, the lateral ventricles’ masks are obtained. We process the masks transform into physical models through 3D printing. From this moment, an object mimicking the lateral ventricles but with a measurable geometry exists in our tangible world. One can accurately calculate the volume of the 3D objects using the WD device presented in Fig. 1.

The Fig. 4 shows the process of 3D modeling and MRI phantom creation for a normal subject and two cases of hydrocephalus. A trained operator manually segments the ventricles from the templates created as explained in Section: “Children’ templates”. We next transform the resulting masks to stereolithography (STL) format^19^. Next, the STL files are loaded in Cura^20^ using a $0.1\;$mm on all axis. Then, we move the models to gcode^21^ format before printing them in a Monoprice Ultimate 3D printer Device using $0.1\;$mm of precision and 25% for structural filling. From this moment, a physical-measurable object exists with dimensions in the real world; however, its form is complex, and deterministic-measuring methods are unpractical. The next step is to determine the volume of the constructed models.

### MRI phantoms, algorithm validation and accurate volume estimation

**Figure 5:**
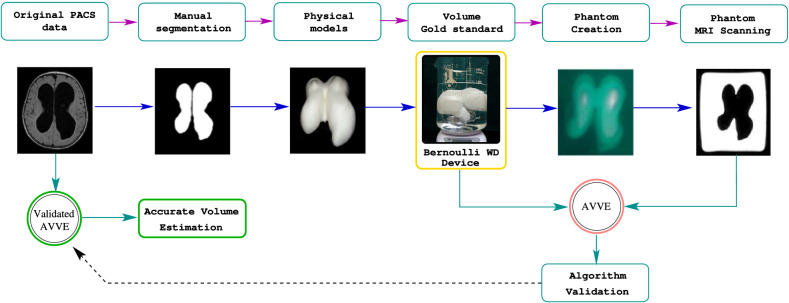
MRI phantoms and validation process. Since the WD device is tuned with marbles in the measurement range, the possible differences between the gold-standard volume and the one yield by the testing algorithm could be attributed entirely to the algorithm. In case the measurements are equal in this stage, the algorithm is certified to measure on patients.

The phantom creation consists of suspending the ventricular 3D models in a solution jelly:water (1 g:3 ml). The inert material of the 3D model surrounded by the watery fixation creates the needed contrast on an MRI scanner from where we obtained the synthetic images (Syim). At this point, one can compare the volumes derived from Syim with those of the physical objects. Such as comparison serves as a validation strategy to certify that volume measuring algorithms—including others than the AVVE—perform with precision. Then, the algorithm is used to measure directly in the original images as it is shown in Fig. 5.

## Supplementary Information

Supplementary Video S1.

Supplementary Legends.

## References

[1] Gerhardt P, Frommhold W (1988). Atlas of Anatomic Correlations in CT and MRI.

[2] Bartels, F. *et al*. Clinical and neuroimaging findings in MOGAD-MRI and OCT. *Clin. Exp. Immunol*. 10.1111/cei.13641. (2021). 10.1111/cei.13641 PMC8561692 34152000

[3] Manganaro L (2017). Fetal mri of the central nervous system: State of the art. Eur. J. Radiol..

[4] Swinburne Nathaniel, C., Bansal Anmol, G., Amit, A., & Doshi Amish, H. Neuroimaging in central nervous system infections. *Curr. Neurol. Neurosci. Rep.* . 10.1007/s11910-017-0756-8 (2017). 10.1007/s11910-017-0756-8 28466277

[5] Tumani, H., Huss, A. & Bachhuber, F. Chapter 2—The cerebrospinal fluid and barriers-anatomic and physiologic considerations. in *Cerebrospinal Fluid in Neurologic Disorders*. Vol. 146. *Handbook of Clinical Neurology*. 21–32. (Deisenhammer, F., Teunissen, C.E. & Tumani, H. eds.). 10.1016/B978-0-12-804279-3.00002-2 (Elsevier, 2018). 10.1016/B978-0-12-804279-3.00002-2 29110772

[6] Weston PG (1915). The cholesterol content of cerebrospinal fluid. J. Med. Res..

[7] Sass LR (2017). A 3D subject-specific model of the spinal subarachnoid space with anatomically realistic ventral and dorsal spinal cord nerve rootlets. Fluids Barriers CNS.

[8] Levi Chazen J (2017). Automated segmentation of MR imaging to determine normative central nervous system cerebrospinal fluid volumes in healthy volunteers. Clin. Imaging.

[9] Brambilla P (2001). Mri study of posterior fossa structures and brain ventricles in bipolar patients. J. Psychiatr. Res..

[10] Punithakumar, L., Noga, M., Ayed, I. B. & Boulanger, P. Right ventricular segmentation in cardiac MRI with moving mesh correspondences. *Comput. Med. Imaging Graph.* **43**, 15–25 (2015). 10.1016/j.compmedimag.2015.01.004 25733395

[11] Yepes-Calderon F, Nelson MD, McComb JG (2018). Automatically measuring brain ventricular volume within PACS using artificial intelligence. PlosOne.

[12] Kim H (2015). Quantitative evaluation of image segmentation incorporating medical consideration functions. Med. Phys..

[13] Yepes-Calderon, F., & Gordon McComb, J. *Manual Segmentation Errors in Medical Imaging. Proposing a Reliable Gold Standard*. 230–241. (Springer, 2019).

[14] Annas, G. J. *Hipaa Regulations—A New Era of Medical-Record Privacy?* (2003). 10.1056/NEJMlim035027 12686707

[15] Avants BB, Tustison NJ, Song G, Cook PA, Klein A, Gee JC (2011). A reproducible evaluation of ants similarity metric performance in brain image registration. NeuroImage.

[16] Smith S (2002). Fast robust automated brain extraction. Hum. Brain Mapp..

[17] Bet2: Mr-based estimation of brain, skull and scalp surfaces. *Hum. Brain Mapp.* **17**, 143–155 (2002).

[18] Hughes SW (2005). Archimedes revisited: A faster, better, cheaper method of accurately measuring the volume of small objects. Phys. Educ..

[19] Stroud I, Xirouchakis P (2000). Stl and extensions. Adv. Eng. Softw..

[20] Dhore, G. *et al*. Exploring 3D printing using Cura: A slicing software. *Int. J. Adv. Sci. Res. Eng. Trends* **5** (2021).

[21] Duong TH (2018). G-code visualization and editing program for inexpensive metal 3D printing. Proc. Manuf..

