# Supplementary material for: Accurate image-based CSF volume calculation of the lateral ventricles
Source: Sci Rep. 2022 Jul 15;12:12115. doi: 10.1038/s41598-022-15995-w (PMC9287564; doi:10.1038/s41598-022-15995-w)
Supplement: Supplementary file 2 — Supplementary Legends. [file 41598_2022_15995_MOESM2_ESM.pdf]

S1_Video_Caption

The video explains the operation of the water displacement device using the schematic in Fig 1. Then, a demonstration with a small marble (9.6 ml) presents evidence of a correct overall operation. In the last part of the video, we extract the volume of a bigger marble (60.8 ml), using a split-screen to display the physical setup, the commands console, and the pulses generation. The scales on the video can not display the extracted volumes with enough precision. However, higher accuracy is accomplished with the sensor's pulsation.
